# The Suture-In-Needle, Closed-Loop Technique for Repositioning a Dislocated Akreos Adapt Intraocular Lens

**DOI:** 10.1155/joph/6846620

**Published:** 2025-03-19

**Authors:** Jingjing Zhang, Fang Liu, Kunkun Zheng, Lei Wan

**Affiliations:** ^1^Eye Institute of Shandong First Medical University, Eye Hospital of Shandong First Medical University (Shandong Eye Hospital), Jinan 250021, China; ^2^State Key Laboratory Cultivation Base, Shandong Key Laboratory of Eye Diseases, Qingdao 266071, China; ^3^School of Ophthalmology, Shandong First Medical University, Jinan 250117, China; ^4^Eye Institute of Shandong First Medical University, Qingdao Eye Hospital of Shandong First Medical University, Qingdao 266071, China

## Abstract

**Purpose:** To evaluate a modified minimally invasive technique for trans-scleral repositioning of dislocated Akreos Adapt intraocular lenses (IOLs) without scleral flaps.

**Methods:** This retrospective case series included 17 eyes with subluxated or dislocated IOLs that underwent repositioning using a suture-in-needle, closed-loop technique. The procedure involved passing an 8-0 polypropylene suture through the IOL's four fenestrated haptics using a bent 30-gauge needle. The exterior suture knot was buried into the sclera without creating scleral flaps or dissecting the conjunctiva. Data on pre- and postoperative intraocular pressure, best-corrected visual acuity, IOL position, corneal endothelial cell counts, and intra-/postoperative complications were collected and analyzed. The follow-up period lasted at least 6 months.

**Results:** All 17 cases demonstrated stable and well-centered IOLs with improved visual acuity. No significant complications, including IOL tilt, decentration, vitreous hemorrhage, hypotony, iris capture, or suture erosion, were observed during the follow-up.

**Conclusion:** The suture-in-needle, closed-loop technique for trans-scleral refixation of dislocated Akreos Adapt IOLs is minimally invasive, achieves excellent anatomical and functional outcomes, and reduces the risk of complications.

## 1. Introduction

Intraocular lens (IOLs) dislocation is a frequent long-term complication following IOL implantation. Treatment options for a dislocated posterior chamber IOL depend on its design and material, with management strategies typically involving either repositioning or replacement [1–6].

The Akreos Adapt IOL (Bausch & Lomb, Laval, Quebec, Canada), a foldable acrylic lens with four points of haptic fixation, is increasingly used because of its clinical and refractive stability for in-the-bag placement or secondary insertion using scleral fixation in the absence of capsular support. In cases of Akreos Adapt IOL dislocation, whether due to loss of zonular stability in the capsular bag or suture failure in scleral-fixated lenses, refixation of the lens is crucial and challenging. While some reports have addressed the treatment of fully dislocated IOLs with four fenestrated haptics, managing such cases remains challenging due to the steep learning curve and technical complexities involved [2, 3].

Herein, we describe a modified and reproducible technique for four-point trans-scleral refixation of Akreos Adapt IOLs using an 8-0 polypropylene and a 30 gauge long needle.

## 2. Materials and Methods

The medical records of all patients with dislocation of the Akreos Adapt IOL (Bausch & Lomb, Laval, Quebec, Canada) who underwent flapless 8–0 polypropylene closed-loop suturing with a 30 gauge needle to achieve four-point IOL refixation at our institution between January 2020 and June 2022 were reviewed. Trans-scleral fixation of Akreos Adapt IOLs was performed as an off-label/unlicensed procedure. This retrospective study followed the tenets of the Declaration of Helsinki and was approved by the Institutional Review Board of Qingdao Eye Hospital of Shandong First Medical University. Written informed consent was obtained from all patients. The inclusion criterion was eyes with dislocated Akreos Adapt IOLs that were either partially subluxated or completely dislocated into the vitreous cavity. The following data were collected from patients' medical records: age, sex, pre- and postoperative intraocular pressure (IOP), best-corrected visual acuity (BCVA), IOL position, corneal endothelial cell counts (Konan Noncon Robo, Konan Medical, Tokyo, Japan), and complications during or after surgery. The minimum follow-up period was 6 months.

IOL tilt and decentration were evaluated using swept-source optical coherence tomography (CASIA2; Tomey Corp., Nagoya, Japan) following previously established methods [7, 8]. The Lens Biometry mode captured 16 images, and the Lens Scan mode captured eight images. The software (Version SS2000) automatically analyzed 3D outlines, tilt, and decentration relative to the corneal vertex axis. Snellen visual acuity was converted to the logarithm of the minimum angle of resolution. Visual acuity of counting fingers was recorded as the logarithm of the minimum angle of resolution 2.0 [9]. Statistical analysis was performed using SPSS software (Version 23.0; ICM Corp., Armonk, NY, USA). Pre- and postoperative BCVA, IOP, and corneal endothelial cell counts were compared using the Student's *t*-test. Statistical significance was set at *p* < 0.05.

### 2.1. Surgical Technique

**Figure 1:**
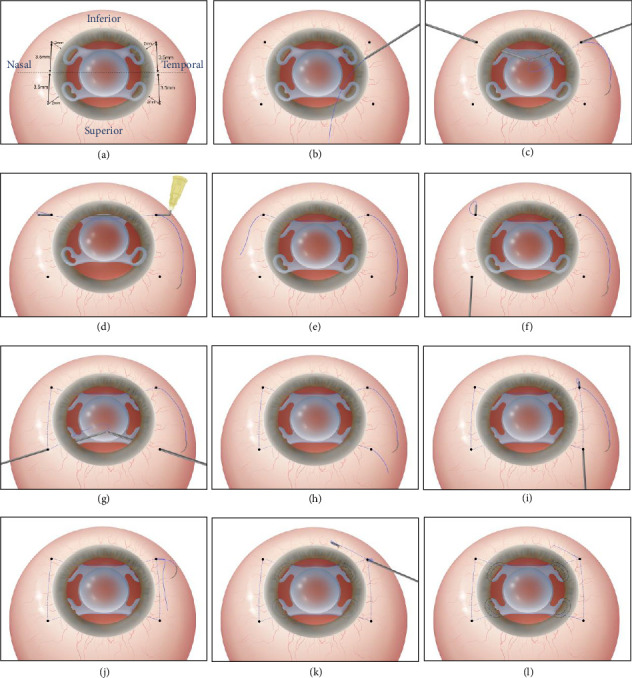
The key surgical procedure diagram. (a) Gentian violet marks four scleral fixation points, and the akreos adapt IOL is placed in the anterior chamber. (b) The 8-0 suture is inserted into a bent 30 gauge needle. (c) The needle is inserted into the posterior chamber, passing through the inferotemporal haptic of the IOL, with its tip docking into a 26 gauge needle 180° away. (d) The needle is guided out of the eye in a needle-to-needle fashion. (e) The suture end is withdrawn, and the needle is retracted. (f) The suture end is passed from the inferonasal to the superonasal subconjunctival fixation points, guided by the 30 gauge needle. (g) The suture is introduced through another IOL eyelet and two additional superior scleral fixation points. (h) The suture has passed through the eye and four IOL eyelets. (i) Thread manipulations are made with a subconjunctival pass from the superotemporal to the inferotemporal scleral fixation points. (j) After adjusting the IOL for centration, the suture ends are tied, followed by an overhand knot. (k) The other end of the suture is loaded into the 30 gauge needle to maintain the tunnel parallel to the limbus. (l) The suture is drawn out to bury the second knot, and the externalized end is trimmed flush with the sclera.

All surgical procedures were performed by the same surgeon (L.W.) under peribulbar anesthesia. The Constellation Vision System (Alcon Laboratories Inc., Duluth, GA, USA) was used to perform 25 gauge pars plana vitrectomy in cases where the dislocated IOL dropped into the vitreous cavity. The basic steps of the technique and the schematic (right eye) are shown in Figure 1.

The horizontal corneal meridian was marked using gentian violet, with the visual axis at the center. Four scleral puncture sites were marked 3.5 mm above and below the horizontal corneal marks, 2 mm from the limbus (Figure 1(a)). Two 1.0 mm incisions were made in the clear cornea at the 10 o'clock and 2 o'clock positions of the corneal limbus. After the anterior chamber was filled with the ophthalmic viscosurgical device, the IOL was gripped with forceps and placed above the iris plane. An 8-0 polypropylene suture (Ethicon, Johnson & Johnson, New Brunswick, NJ) was cut at its midpoint; one end was inserted approximately 10 mm inside the hollow lumen of a curved 25 mm, 30 gauge needle attached to a syringe (Figure 1(b)). The needle with the loaded 8-0 polypropylene suture was passed into the posterior chamber through the sclera, 2 mm behind the corneal limbus. It was then passed through an eyelet on the inferotemporal haptic of the IOL and dragged across the globe using a 26 gauge needle, passed through another eyelet 180° away on the inferonasal haptic of the IOL in a needle-to-needle manner (Figure 1(c)). The 8-0 polypropylene suture was passed parallel to the horizontal meridian from the inferotemporal to the inferonasal scleral bed through the eye and two IOL eyelets (Figure 1(d)). Forceps were used to grasp the end of the 8-0 polypropylene suture in the lumen of the 30 gauge needle (Figure 1(e)). The 30 gauge needle was retrieved from the eye and passed intrasclerally or subconjunctivally, parallel to the limbus, from the inferonasal to the superonasal fixation points. The end of the 8-0 polypropylene suture was placed in the lumen of the 30 gauge needle and returned in the opposite direction (Figure 1(f)). The procedure was repeated when introducing an 8-0 polypropylene suture into the eye and additional IOL eyelets via two additional upper scleral fixation points (Figures 1(g) and 1(h)). Identical manipulations of the suture, passing from the superotemporal to the inferotemporal scleral fixation points, were performed (Figure 1(i)). Suture tension was adjusted to achieve optimal IOL centration. The bilateral suture ends were knotted in the sclera (Figure 1(j)). A second overhand knot was made approximately 4 mm from the first knot. One suture end was cut approximately 2 mm from the second knot. The other end of the suture was placed in the lumen of the bent 30 gauge needle, adapted for creating a 4–5 mm limbus-parallel tunnel in the sclera. The suture was pulled to bury the second knot in the scleral tunnel (Figure 1(k)). Finally, the external end of the suture was cut flush with the scleral surface (Figure 1(l)). The conjunctival incisions were not sutured.

## 3. Results

The suture-in-needle, closed-loop technique of trans-scleral refixation of dislocated Akreos Adapt IOLs was employed in 17 eyes of 17 patients (13 men and 4 women) aged 27–79 (mean ± standard deviation: 58.6 ± 17.7) years with the follow-up period of 13.6 ± 7.8 months (range 6–24 months). The causes of IOL dislocation in this cohort were as follows: nine eyes experienced dislocation after vitrectomy, three eyes resulted from trauma, one was associated with retinitis pigmentosa, and four eyes had no identifiable cause. All patients had successful scleral refixation of the dislocated IOL.

All patients exhibited improved visual acuity. The preoperative BCVA ranged from 20/2000 to 20/20. The final postoperatively BCVA ranged from 20/63 to 20/20. The logMAR BCVA was ameliorated from the preoperative 1.51 ± 0.59 to postoperative 0.22 ± 0.23 (*p* < 0.001). There was no significant difference between the mean preoperative IOP (16.1 ± 2.9 mmHg) and mean postoperative IOP (15.9 ± 2.5 mmHg) at the final follow-up (*p*=0.602). The mean corneal endothelial cell density decreased from 2391 ± 578 to 2259 ± 506 cells/mm^2^ (*p*=0.208). At the final follow-up, the mean IOL tilt in 17 eyes was 3.13° ± 2.12°, and the mean decentration was 0.34 ± 0.21 mm.

**Figure 2:**
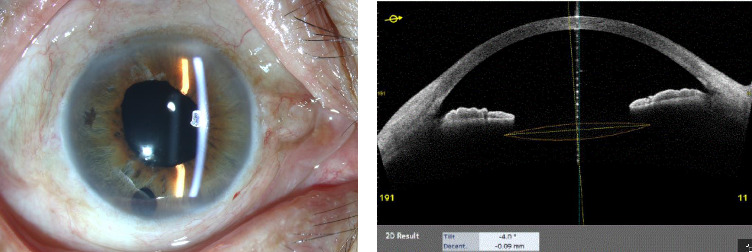
Presentation 6 months postoperatively. (a) Slit-lamp microscopy image showing trimmed suture ends without erosion or exposure. (b) The optical coherence tomography image shows no decentration or tilt of the intraocular lens.

There were no intraoperative complications. Mild vitreous hemorrhage observed in one eye resolved within 1 week. The intraocular pressure was transiently elevated (> 21 mmHg) in one eye 2–3 weeks postoperatively and normalized 1 month postoperatively without further intervention. Hypotony (≤ 6 mmHg) occurred in two eyes, but both recovered within 1 week of the surgery without further surgical intervention. The suture ends were not exposed or shed after trimming (Figure 2(a)). The IOLs remained stable and centered throughout the follow-up (Figure 2(b)).

No other complications were observed, such as cystoid macular edema, persistent uveitis, iris capture of the IOL, and choroidal or retinal detachment.

## 4. Discussion

IOL dislocation is a common long-term complication of IOL implantation, and its risk factors usually include retinitis pigmentosa, history of vitrectomy surgery, trauma, and high myopia, with an incidence rate of approximately 0.5%–3% [10]. In our cohort, the primary cause of IOL dislocation was a history of vitrectomy surgery. Apart from high myopia, the other risk factors were generally consistent with those reported in the literature. The treatment strategies for IOL dislocation can be customized depending on the types and status of dislocation [11, 12]. Refixation is less invasive than conventional IOL replacement, avoiding a large corneal/scleral incision during IOL exchange. The surgical technique in this study required the formation of only two 1-mm corneal incisions to pass sutures through the eyelet of IOLs. This minimal corneal incision substantially reduced the incidence of astigmatism commonly associated with corneal or scleral incisions during IOL replacement procedures.

The Akreos Adapt posterior chamber IOL has minimal tilt risk and ideal stability because it has four haptics supporting the lens. The final follow-up of the study reported a mean IOL tilt of 3.13° ± 2.12° and a mean decentration of 0.34 ± 0.21 mm across 17 eyes. This degree of tilt and decentration is considered clinically acceptable, as it does not significantly impact visual quality. It has gained popularity due to its clinical stability for secondary implantation in the bag placement or scleral fixation without capsular support [13–16]. Cases of dislocated IOLs with four-looped haptics present a unique challenge. Recently, Fan and Smiddy described a suitable and efficient surgical technique for intrascleral suture refixation of the Akreos AO60 lens without externalizing the IOL haptics [2]. However, their technology still requires extensive conjunctival dissections and scleral flaps. This study presents a modified surgical approach using a single-string, closed-loop technique to refixation dislocated Akreos four-haptic IOLs, eliminating the need for scleral flaps, pockets, or grooves. Inappropriate rotation or movement of IOLs dislocated into the vitreous cavity can result in retinal damage, potentially leading to retinal tears or detachment. Contrary to a previous study [2], the threading process of the IOL loop in this study was primarily conducted at the iris level, significantly minimizing the risk of retinal damage associated with surgical procedures. All patients in this study experienced satisfactory surgical outcomes, with no related complications reported.

The suture-in-needle technique and trans-scleral suture fixation of a posterior chamber IOL were first described in 1986 [17]. Nevertheless, suture breakage and degradation over time remain significant problems. In one study, the incidence of breakage of 10-0 polypropylene sutures was 27.9% at 6 years post-IOL implantation [18]. Many surgeons currently use Gore-Tex sutures for scleral fixation IOLs due to their resistance to degradation, high tensile strength, and minimal inflammatory response [19, 20]. Although these sutures have not yet received specific approval for ophthalmic use, they are widely regarded as a safe and reliable option. In recent years, 8-0 polypropylene sutures have also gained popularity due to their resistance to degradation and high tensile strength [3, 21, 22]. Both Gore-Tex and 8-0 polypropylene sutures have their advantages and limitations. As with all suture-based scleral fixation techniques, these two sutures may lead to complications such as scleritis and cystoid macular edema. However, no studies have yet compared the incidence of these complications between Gore-Tex and 8-0 polypropylene sutures [20, 23]. When making clinical decisions, it is essential to consider the advantages, disadvantages, and clinical availability of each suture type.

A 25 mm long 30 gauge needle on a syringe instead of a needle holder is more operable than the previous 10-0 suture with an attached straight needle [24]. In addition, the 30 gauge-guiding needle can assist various sizes of sutures to pass through the looped IOL haptics, providing more options for surgeons. This assisted suturing technique for introducing fixation sutures in the eye and looping IOL eyelets is similar to previously described [3]. Contrarily, we implemented a closed continuous loop technology that offers several advantages. First, it requires only a single knot, thereby minimizing the necessity for multiple knots in processing operations. Second, the two lines traversing the haptic hole in the eye experience uniform stress, enhancing the IOLs' stability.

Externalizing the intraocular suture is a key step in the fixation technique of trans-scleral suture fixation of a posterior chamber IOL. Fine microscopic instruments can better assist in externalizing intraocular sutures, such as 25 gauge forceps-needle and 27 gauge forceps [25, 26]. However, these instruments are often expensive due to limited production and supply and are difficult to obtain. As initially described by Jin et al. [27], the needle-to-needle technique for suture externalization employed in this study offers several advantages. These include a simplified surgical process, good maneuverability, and the elimination of haptic externalization. In addition, all required equipment is both affordable and readily available. Ab externo scleral suture loop fixation is a widely accepted technique for repositioning dislocated in-the-bag IOLs [28]. However, it involves repeatedly passing a long needle through the eyeball and requires careful control to prevent intraocular tissue damage. The suture snare is a straightforward solution that resolves the problem of unsuitable needles, eliminating the need for a large sclerostomy and expensive tools [29, 30]. Applying suture snare technology for displaced Akreos Adapt IOLs and its safety and efficacy evaluation presents a promising research direction for our team.

In cases of external suture-end processing, we employed a previously reported technique [31]. The modified technique is less traumatic because it avoids a sizable conjunctival incision and involves the creation of scleral flaps, pockets, or grooves that reduce surgical complexity and shorten the operative time. The procedure potentially facilitates passing subconjunctival or intrascleral sutures when noticeable conjunctival or scleral tissue scarring occurs. This flapless technique may be an alternative for patients who have undergone trabeculectomy. The surgeon does not require additional surgical instruments or devices, except for a 30 gauge needle, which is readily available and can guide the suture through the subconjunctival or scleral layers, reducing late complications, such as postoperative suture exposure. This study has limitations, including its small sample size, retrospective nature, the lack of a comparison cohort, and an insufficient follow-up period. Studies with larger sample sizes and more extended follow-up periods should be conducted to determine whether visual acuity, complications, and overall safety are stable in the long term.

## 5. Conclusions

We present a safe, effective, minimally invasive, and reliable flapless technique for four-point trans-scleral refixation of dislocated Akreos Adapt IOLs.

## Data Availability

The data supporting this study's findings are available from the corresponding author upon reasonable request.
